# Retraction: Social and economic factors responsible for environmental performance: A global analysis

**DOI:** 10.1371/journal.pone.0311387

**Published:** 2024-11-11

**Authors:** The PLOS ONE Editors

The *PLOS ONE* Editors retract this article [1] because it was identified as one of a series of submissions for which we have concerns about authorship, ethics approval, integrity of the underlying data, reliability of the published results, and peer review. We regret that the issues were not identified prior to the article’s publication.

During post-publication discussions about these concerns, Peter W. Cardon (PWC) informed PLOS that they did not contribute to this article and were not aware that they had been listed as a co-author.

GRM did not agree with the retraction. PWC responded but expressed neither agreement nor disagreement with the editorial decision. CW and JL either did not respond directly or could not be reached.
